# Supplementary material for: Integrated reconstructive spectrometer with programmable photonic circuits
Source: Nat Commun. 2023 Oct 11;14:6376. doi: 10.1038/s41467-023-42197-3 (PMC10567699; doi:10.1038/s41467-023-42197-3)
Supplement: Supplementary file 1 — Supplementary Information [file 41467_2023_42197_MOESM1_ESM.pdf]

Supplementary Information

Integrated reconstructive spectrometer with programmable photonic circuits

Chunhui Yao[1], Kangning Xu[2], Wanlu Zhang[1], Minjia Chen[1], Qixiang Cheng[1,2*], Richard Penty[1]

1. Centre for Photonic Systems, Electrical Engineering Division, Department of Engineering, University of Cambridge, Cambridge, CB3 0FA, UK

2. GlitterinTech Limited, Xuzhou, 221000, China

**Corresponding author: qc223@cam.ac.uk*

S1. Further discussions regarding the computational spectrum reconstruction

Recovering the spectrum signal $\Phi \in \mathbb{R}^N$ based on Eq. (2) poses an underdetermined inverse problem, as $M \ll N$. Conventional $\ell_2 - norm$ minimization method shown in Eq. (3), i.e., the least square method, is inadequate for solving this problem since it necessitates a full column rank for the transmission matrix *T*. Consequently, various regularization strategies, such as the modified Tikhonov regularization (see Eq. (4)), are proposed to reduce the ill-conditioning of underdetermined inverse problems. To gain a deeper insight, here we adopt the compressive sensing (CS) theories in this context, as they have been proven as effective approaches for tackling such underdetermined problems[1]. Specifically, the CS theories assert that most practical incident spectrum signals are either sparse (e.g., the narrowband laser signals) or compressible, such that they can be sparsely represented in a specific orthonormal basis Ψ, as[2]:

$$\Phi = \Psi s \tag{S1}$$

where s is an $N \times 1$ vector in $\mathbb{R}^N$, which only consists of K non-zero elements, $K \ll N$. The recovery of these sparse signals can be expressed as $\ell_0 - norm$ minimization:

$$\hat{s} = arg \min_{s \in \mathbb{R}^N} \|s\|_0, \ \ subject\ to\ I = \Theta s \tag{S2}$$

where $\Theta \coloneqq T\Psi$. Solving Eq. (S2), however, suffer from combinatorial computational complexity. Thus, $\ell_1 - norm$ minimization, as a convex optimization approximation of the $\ell_0 - norm$ minimization, is required to be introduced, as:

$$\hat{s} = arg \min_{s \in \mathbb{R}^N} \|s\|_1, \ \ subject\ to\ I = \Theta \mathrm{s} \tag{S3}$$

By solving such convex optimization problem with a well-designed transmission matrix, the spectrum Φ can be accurately reconstructed with high resolution through limited sampling times following a logarithmic relationship[3], i.e., $M \sim O(C\log N)$, where C is a constant that relates to the level of sparsity K, the reconstruction algorithm itself, and the mutual coherence of Θ, which is defined as:

$$\mu(\Theta) = \max_{i \neq j} \frac{|\langle \theta_i, \theta_j \rangle|}{\|\theta_i\|_2 \|\theta_j\|_2} \tag{S4}$$

where θ_i is the column vector of Θ. Note that the estimation of the minimum sampling number M is primarily influenced by the signal sparsity K and the $\mu(\Theta)$ determined by the transmission matrix's structure, while the selection of different reconstruction algorithms mainly impact the noise tolerance and time complexity[4]. In this paper, all the spectrum reconstruction simulations are conducted via the CVX convex optimization algorithm[5].

To further quantify the relationship of $M \sim O(C\log N)$, we simulate the ideal transmission matrices and investigate the reconstruction performance (i.e. its resolution and accuracy) under varying numbers of sampling

channels. As discussed in the main body text, a high-performance transmission matrix should have a small auto-correlation width δλ and low cross-correlation between channels. This indicates that for the most ideal transmission matrix, its auto-correlation function $C(\Delta\lambda)$ should be a Dirac-delta function as it features the smallest possible auto-correlation width δλ, i.e., $\lambda_{bandwidth}/N$, while the cross-correlation should approach zero. Mathematically, the elements of such matrix (i.e., with Dirac-delta auto-correlation function and zero cross-correlation) ought to be independent identically distributed (i.i.d.) random variables, which is, however, unattainable for any physical optical structures. Hence, to generate a quasi-ideal transmission matrix that is achievable, we first create an i.i.d. random matrix that follows $\lambda_{bandwidth}/N$ = 0.2 nm, and then fit the discrete elements in each row (i.e., each sampling channel) using cubic spline interpolation to make them continuous. By this manner, transmission matrices with a consistent auto-correlation width δλ of 0.2nm are obtained, as illustrated by Fig. S1(a-b). Utilizing these matrices, we explore the relationship between the sampling channel number M and the resolution/spectral pixel N, as depicted in Fig. 1(a). Here, $\Theta = T$ since the dual-peak signal is originally sparse. As the spectral pixel N grows larger, the value of the auto-correlation function with a smaller lag $\Delta\lambda$, i.e., $C(\lambda_{bandwidth}/N)$, gradually approaches 1, as shown by the inset of Fig. S1(b). This implies that the adjacent column vectors in such a matrix become nearly identical, i.e., $\theta_i \approx \theta_{i+1}$, leading to a high mutual coherence $\mu(\Theta)$. Therefore, when N is considerably large, the changes in the constant C become more significant given the high value of $\mu(\Theta)$, so that even a small increment in N would result in a substantial increase in M, explaining the stepwise curve seen in Fig. 1(a). On the other hand, we reveal the enhancement in reconstruction accuracy with larger sampling number M by solving the broadband ASE spectrum from an EDFA, as shown by Fig. 1(b). Note

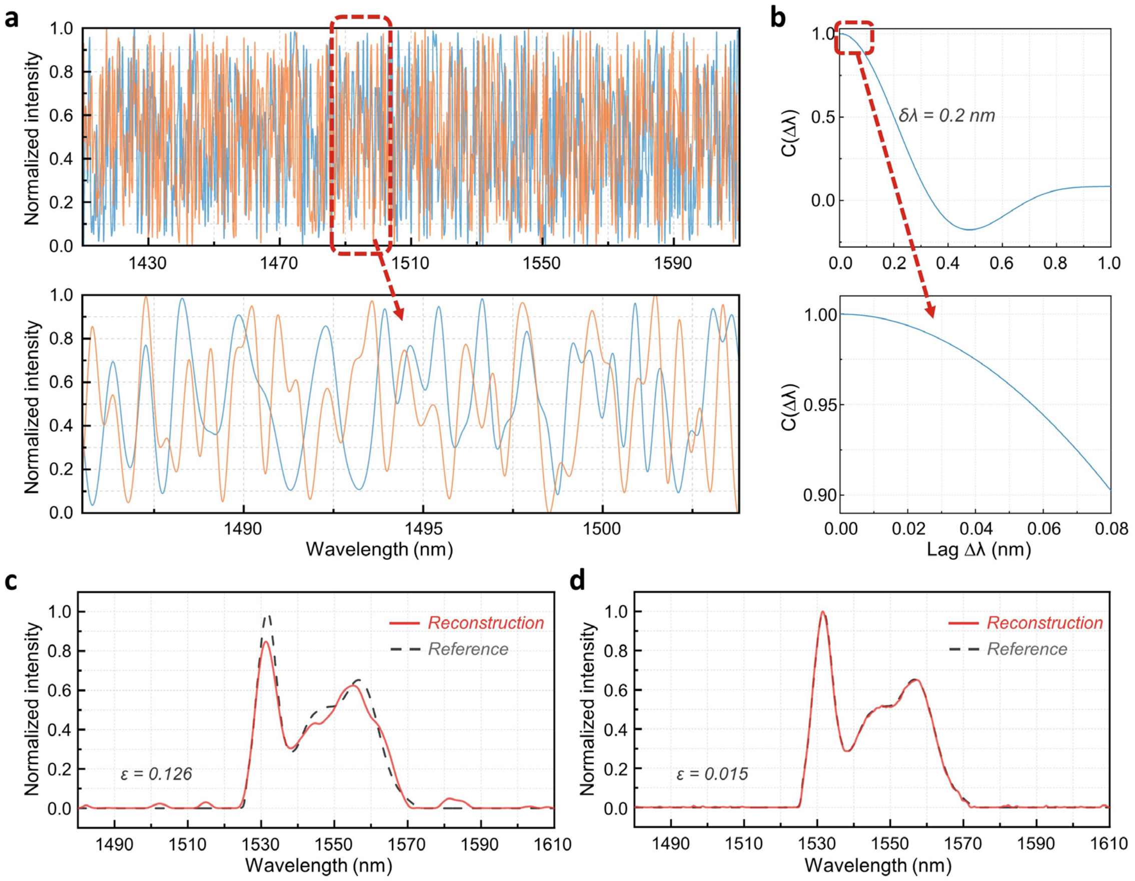

Figure S1 | Simulation results based on ideal transmission matrices. (a) Transmission spectra of two exemplary sampling channels from a randomly generated ideal transmission matrix. The inset emphasizes the spectral randomness. (b) The calculated auto-correlation function $C(\Delta\lambda)$ of the ideal transmission matrix, with a auto-corrlation width δλ of 0.2 nm. The inset shows that the value of $C(\lambda_{bandwidth}/N)$ approaches 1, when the spectral pixel N is relatively large. (c-d) Reconstructed spectra of a broadband signal using 40 and 300 sampling channels, repsectively, showing the improvement in reconstruction accuracy.

that in our simulations, we include additive white gaussian noise with a signal-to-noise ratio of 30 dB to emulate realistic conditions. Figures S1(c-d) displays the reconstructed ASE spectrum using 40 and 300 sampling channels, respectively. It is evident that a larger sampling number effectively reduces the relative error (from 0.126 down to 0.015).

S2. Geometry design, simulations, and further experimental results

To facilitate high-performance channel spectral responses with small auto- and cross-correlation, the proposed cascading system is optimized on the basis of Eq. (7). Here, we conclude the two key design criteria. First, a small FSR at each MZI stage is desired in order to induce rapid fluctuations in the wavelength domain for high sampling

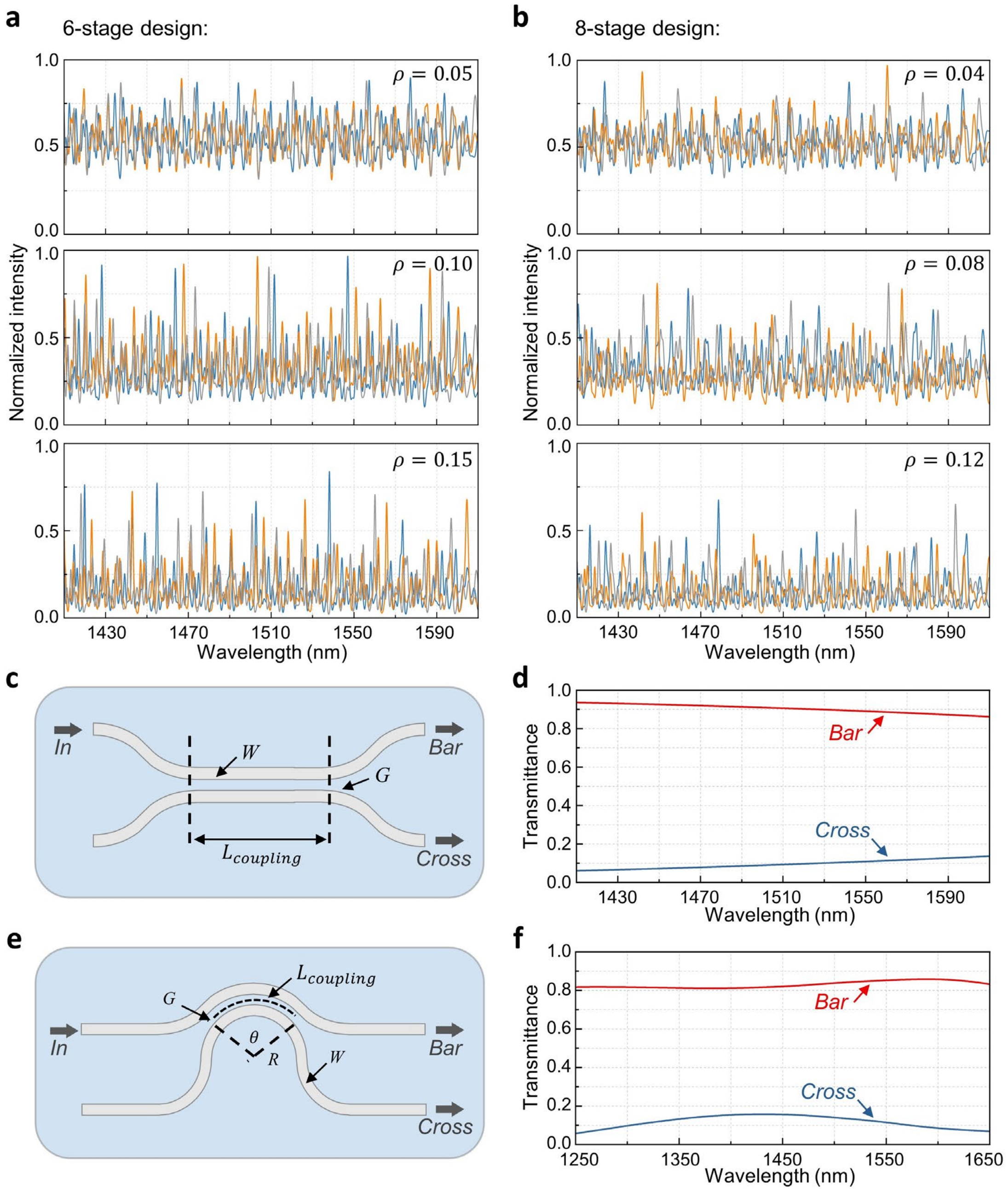

Figure S2 | Parameter design and simulation. (a-b) Examples of the simulated transmission spectra for a 6-stage and an 8-stage programmable spectrometer with different power splitting ratio ρ of the MZIs, respectively. (c) Schematic of a symmetric DC. (d) Simulated wavelength dependence of the transmittance of the optimized symmetric DC. (e) Schematic of a curved DC. (f) Simulated wavelength dependence of the transmittance of the optimized curved DC.

efficiency. Meanwhile, the FSRs of the different MZIs must vary with each other, enabling overlaid transmission spectra with sufficient spectral randomness. In our case, the FSR of each MZI is designed to gradually decrease from approximately 9 nm to 2 nm over the stages, which corresponds to the arm length difference ΔL_i increasing from about 135 μm to 605 μm. On the other hand, the ER of each MZI (determined by the coupler splitting ratio ρ) is desired to have a modest value to maximize the intensity contrast in the overlaid spectra (i.e., the range of spectral fluctuations) while minimizing the filtering loss. For example, Fig. S2(a-b) displays several exemplary transmission spectra for a 6-stage design and an 8-stage design, each with varying levels of ρ. As can be seen, a ρ of around 0.10 for the 6-stage design and 0.08 for the 8-stage design is mostly preferred, as they allow the overlaid transmission intensity to fully fluctuate in between 0 and 1.0. In contrast, a larger or smaller value of ρ can still create the pseudo-random spectral fluctuations, though with slightly compromised fluctuation range or excess loss. Following these criteria, we thereby simulate the channel spectral responses of the spectrometer with different numbers of MZI stages N_{stage} and phase tuning state per stage P_{state} using the Lumerical INTERCONNECT, as displayed in Fig. 2(c-e).

In the fabricated device, we employ a symmetrical directional coupler (DC) to realize the desired power splitting ratio, as shown by Fig. S2(c). Lumerical FDTD is used to perform the device simulations. By fine-tuning the geometric parameters of DC, such as its waveguide width W, the length of coupling region $L_{coupling}$, and the gap, G, the power splitting ratio can be maintained consistently over a broad bandwidth. Specifically, for the 6-stage spectrometer deployed on our SiN platform, the optimized design parameters include: $L_{coupling}$ = 500 nm, W = 1200 nm, and G = 450 nm. Figure S2(d) presents the FDTD simulated power transmittance at various output ports as a function of the wavelength, revealing a stable splitting ratio ρ of around 0.1 that varies slightly from around 0.07 to 0.13 across a spectral range exceeding 200 nm. To further broaden such bandwidth, dispersion-engineered waveguide components can be utilized, such as the curved DC[6], as shown by Figure S2(e). Similarly, we optimize the geometry of a curved DC based on our SiN platform and determine the optimal parameters as: inner radius R = 60 μm, $L_{coupling}$ = 22.5 μm (i.e., the coupling angle θ = 21.5°), W = 900 nm, and G = 300 nm. Figure S2(f) shows the simulated port transmittances across a 400 nm bandwidth from 1250 nm to 1650 nm. It can be seen that the splitting ratio ρ remains consistently around 0.1 (between 0.06 and 0.15).

To facilitate precise phase tuning of each MZI stage, we conduct calibration processes by launching a laser signal into the cascaded system and sweeping the driving power of each phase shifter individually, while monitoring the output optical power. As an example, the inset in Fig. S3 presents the measured optical power in

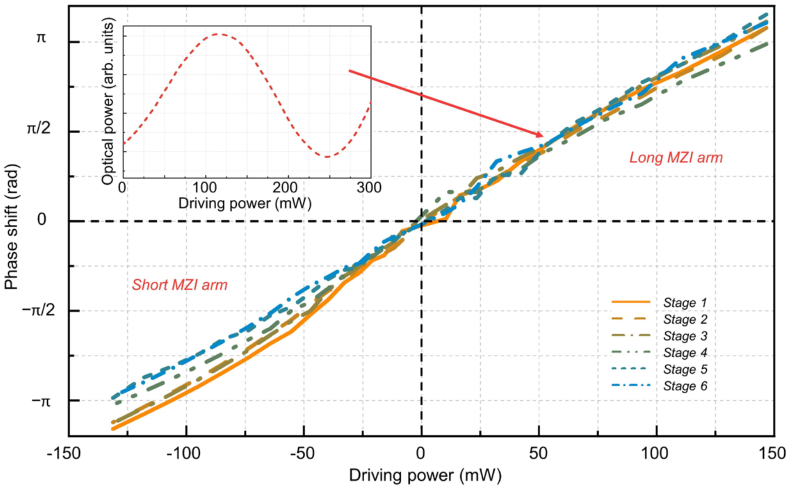

Figure S3 | Thermal phase tuning of the unbalanced MZIs. The phase shift as a function of the driving power across all 6 MZI stages. Note that here the driving power applied to the long arm of the unbalanced MZIs is denoted as positive, leading to a resultant positive phase shift, and vice versa. As an example, the inset shows the measured optical power as a function of the driving power applied to the phase shifter located on the long arm of the first MZI stage.

relation to the driving power applied to the heater on the long arm of the first MZI stage. The smooth sinusoidal curve not only indicates the phase shift, but also confirms the absence of thermal crosstalk between stages. Accordingly, we determine the relationship between the phase shift and driving power across all 6 MZI stages, as shown in Fig. S3. For illustrative purposes, we refer to the driving power applied on the long arm of the unbalanced MZIs as positive, which correlates with a positive phase shift, and vice versa. It can be seen that the complementary phase shifter pair on both MZI arms realizes the phase modulation from $-\pi$ to $+\pi$, with a thermal efficiency of around 42 mW/rad. Hence, in our experiment, the average power is about 350 mW as the phase shifters are set to induce phase shifts of either $-2\pi/3$, 0, or $2\pi/3$. Such thermal efficiency can be notably enhanced by incorporating deep trenches or undercuts adjacent to the waveguide[7]. Meanwhile, the implementation of our design on a Si platform could also significantly elevate the thermal efficiency due to its over 10-times superior thermo-optic coefficient.

S3. Further exploration of device performance

To determine the actual resolution of the fabricated 6-stage RS design with 729 programmed sampling channels, we continue the convergence test through simulations by reconstructing dual-peak inputs with smaller spectral

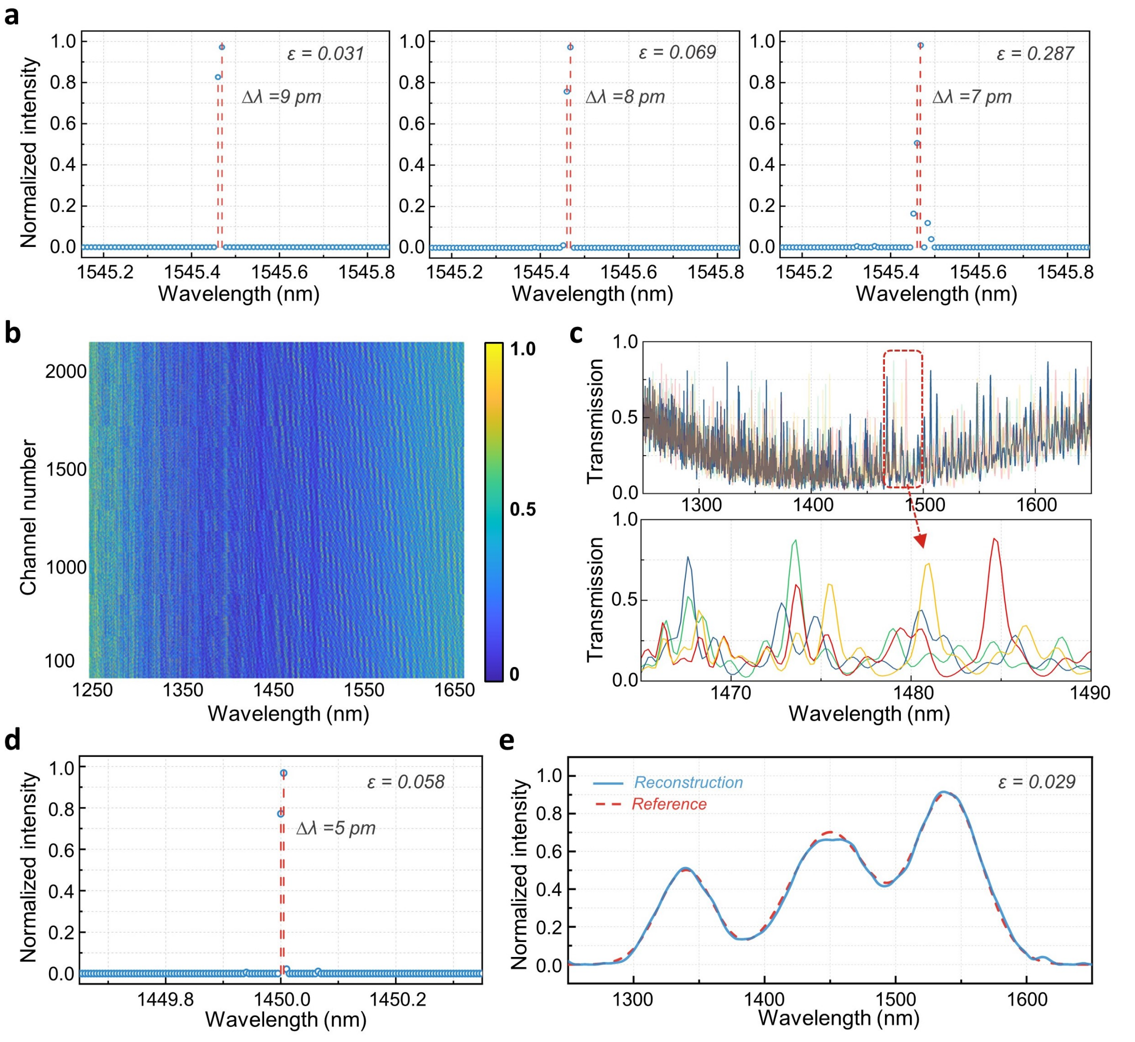

Figure S4 | Simulated spectrum reconstruction based on a 6-stage design with curved DCs. (a) Reconstructed spectra for dual spectral lines with different spectral spacings, using the measured 729 sampling channels. (b) Normalized transmission matrix with 2304 channels. (c) The transmission spectra of several representative sampling channels, showing the pseudo-random spectral fluctuation over the 400 nm bandwidth. (d) Simulated spectrum reconstruction for a dual-peak input with a spectral spacing of 5 pm, using the simulated 2304 channels. (e) Simulated reconstruction for a broadband, continuous spectrum.

spacings. Note that in our simulations, an equivalent level of measurement noise as observed during the experiments is taken into account. Figure S4(a) displays the reconstructed dual-peak signals. As can be seen, a dual-peak input with an 8 pm spectral spacing can still be resolved with good accuracy (relative error ε of 0.069), thereby indicating a resolution of 8 pm.

Moreover, to explore the performance scalability of our design, we investigate a same 6-stage design based on the curved DC designs (as shown in Fig. S2(e-f)). The programmed sampling channel number is also increased to 2304 by adjusting the phase tuning state P_{state} of the first four stages from 3 to 4 (i.e., $4^4 \times 3^2 = 2304$ channels). Figure S4(b-c) shows the simulated transmission matrix and a few representative sampling channels, respectively. It can be seen that pseudo-random spectral fluctuations are well maintained over the 400 nm wavelength range. Figure S4(d) presents the reconstruction result for the input of dual spectral lines with a spacing of 5 pm. The well-distinguished peak intensities thus illustrate a resolution of 5 pm. The reconstruction of a broadband continuous spectrum is also simulated, as depicted in Fig. S4(e). The spectral features are well recovered with a relative error ε of 0.029. These results demonstrate that our device performance could be readily enhanced to reach a < 5 pm resolution with > 400 nm bandwidth or even higher.

S4. Experimental set-up and electrical control

Figure S5 illustrates the experimental set-up and electrical control system used for the spectrometer calibration and testing. For calibration, we launch a broadband ASE source to the chip and use a commercial benchtop optical spectral analyzer (YOKAGAWA AQ6370D) to measure the transmission spectra of different sampling channels. After that, the input light source is switched to the unknown narrowband, broadband or hybrid signals, while a photodiode is used to record the real-time output power intensities under different sampling channels. Here, an electrical control system based on a microcontroller unit (MCU) is used to automatically configure the bias voltages for different sampling channels in temporal and collect the real-time photodiode data. Specifically, the MCU is programmed to transmit the pre-calibrated voltage look-up table to a multi-channel digital-to-analogue converter (DAC, AD5370), generating analog electrical signals. These signals are then amplified by a customized driving board, enabling the phase manipulation of the spectrometer. Simultaneously, the photodiode's output signals are sampled by an analogue-to-digital converter (ADC) embedded in the MCU. Experimentally, such system achieves a sampling speed of less than 1 ms per channel, allowing for the measurement of 729 channels in under 0.8 seconds. We attribute the primary speed bottleneck to the limited data transmission rate (750 kb/s) between the MCU and the DAC, which could be effectively addressed by upgrading our MCU to more advanced

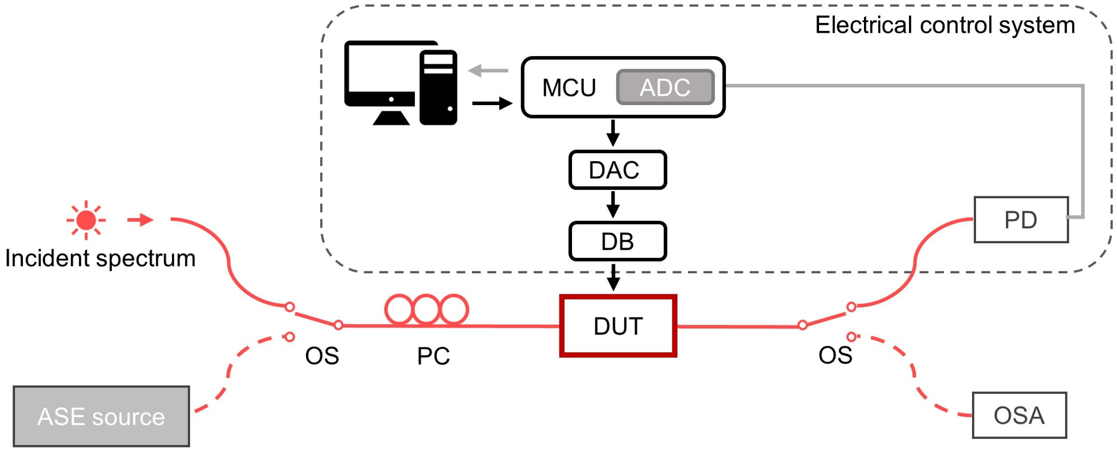

Figure S5 | Experimental set-up and electrical control system for the device calibration and testing. OS: optical switch. PC: polarization controller. DUT: device under test. MCU: microcontroller unit. ADC: analogue-to digital converter. DB: driving board. DAC: digital-to-analogue converter. PD: photodiode. OSA: optical spectrum analyzer.

modules or FPGAs and implementing higher data rate electrical interconnections.

S5. Device thermal robustness

As illustrated in Fig. 4(g), the channel spectral responses exhibit a redshift in relation to the rise in temperature, occurring at a rate of about 15.5 pm per degree. Accordingly, we model the transmission matrices under different temperature variations, and calculate the corresponding output optical power intensities at all sampling channels using a narrowband laser signal. Based on these power intensities, we then use the transmission matrix obtained with no temperature variation to proceed the spectrum reconstructions. As shown by Fig. S6, even with temperature variations of up to ±0.9 °C, the input signal can still be recovered to the accuracy of the spectrometer resolution, i.e., with the offset of center wavelength remaining within ±10 pm.

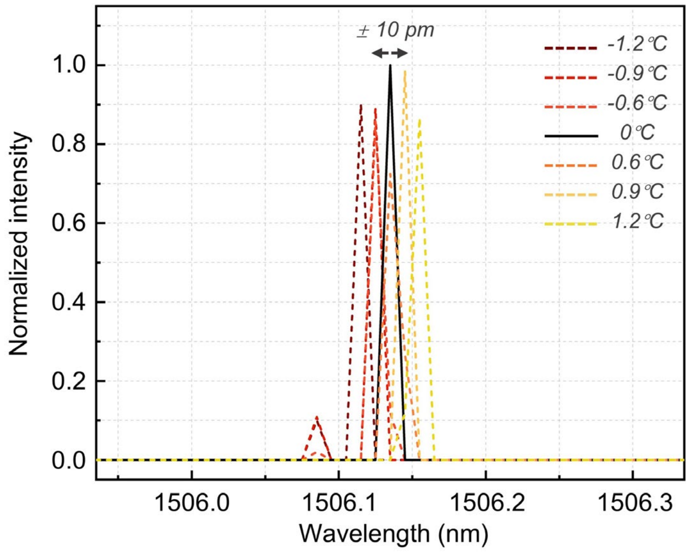

Figure S6 | Device thermal stability. Reconstructed spectra of a narrowband laser signal at different temperatures, showing a good tolerance to temperature variations up to ± 0.9 °C.

S6. Further performance comparison

To further highlight the strengths of our device, we conduct a more comprehensive performance comparison with several representative competitors[8–15], as listed in Table S1. It can be seen that our spectrometer features an outstanding thermal robustness, particularly when compared to other spectrometer designs with high resolutions.

Table S1. Performance comparison with some representative competitor approaches

Ref	Scheme	Resolution (nm)	Bandwidth (nm)	Thermal stability	Sampling time	CMOS compatible
8	Disorder media	0.75	25	±4°C	One-shot	Yes
9	Nanowire	5	250	N.M.	One-shot	No
10	Quantum dot	2-3	300	N.M.	One-shot	No
11	Micro disk	0.2	20	~±0.12°C	N.M.	Yes
12	Multimode ring	0.08	100	±0.014°C	375 s	Yes
13	AWG + ring	0.1	27	~±0.67°C	N.M.	Yes
14	Chirped grating	0.3	70	N.M.	One-shot	Yes
15	Spatial FT	0.0011	6.2	N.M.	One-shot	Yes
This work	Programmable photonics	0.01	200	±0.9°C	<0.8 s	Yes

*N.M.: Not mentioned.

Additionally, our design also enjoys a short sampling time and the CMOS compatibility for massive production, making it well-suited for the emerging demands of cost-effective in-situ spectroscopic devices.

Supplementary References

1. Ye, Y., Zhang, J., Liu, D. & Yang, Y. Research on a Spectral Reconstruction Method with Noise Tolerance. *Curr. Opt. Photonics.* **5**, 562–575 (2021).
2. Kutyniok, G. Theory and applications of compressed sensing. *GAMM-Mitteilungen* **36**, 79–101 (2013).
3. Rani, M., Dhok, S. B. & Deshmukh, R. B. A Systematic Review of Compressive Sensing: Concepts, Implementations and Applications. *IEEE Access* **6**, 4875–4894 (2018).
4. Fountoulakis, K., Gondzio, J. & Zhlobich, P. Matrix-free interior point method for compressed sensing problems. *Math. Prog. Comp.* **6**, 1–31 (2014).
5. Grant, M. C. & Boyd, S. P. CVX: matlab software for disciplined convex programming, version 2.2. http://cvxr.com/cvx. (2020).
6. Morino, H., Maruyama, T. & Iiyama, K. Reduction of Wavelength Dependence of Coupling Characteristics Using Si Optical Waveguide Curved Directional Coupler. *J. Lightwave Technol.* **32**, 2188–2192 (2014).
7. De, S., Das, R., Varshney, R. K. & Schneider, T. Design and Simulation of Thermo-Optic Phase Shifters With Low Thermal Crosstalk for Dense Photonic Integration. *IEEE Access* **8**, 141632–141640 (2020).
8. Redding, B. Compact spectrometer based on a disordered photonic chip. *Nat. Photon.* **7**, 6 (2013).
9. Yang, Z. *et al.* Single-nanowire spectrometers. *Science* **365**, 1017–1020 (2019).
10. Bao, J. & Bawendi, M. G. A colloidal quantum dot spectrometer. *Nature* **523**, 67–70 (2015).
11. Sun, C. *et al.* Scalable On-Chip Microdisk Resonator Spectrometer. *Laser Photonics Rev.*, 2200792 (2023).
12. Xu, H., Qin, Y., Hu, G. & Tsang, H. K. Integrated Single-Resonator Spectrometer beyond the Free-Spectral-Range Limit. *ACS Photonics* **10**, 654–666 (2023).
13. Zheng, S. *et al.* A Single-Chip Integrated Spectrometer via Tunable Microring Resonator Array. *IEEE Photonics J.* **11**, 1–9 (2019).
14. Nezhadbadeh, S. *et al.* Chirped-grating spectrometer-on-a-chip. *Opt. Express* **28**, 24501–24510 (2020).
15. Paudel, U. & Rose, T. Ultra-high resolution and broadband chip-scale speckle enhanced Fourier-transform spectrometer. *Opt. Express* **28**, 16469 (2020).
